# Neural Networks to Predict Radiographic Brain Injury in Pediatric Patients Treated with Extracorporeal Membrane Oxygenation

**DOI:** 10.3390/jcm9092718

**Published:** 2020-08-22

**Authors:** Neel Shah, Abdelaziz Farhat, Jefferson Tweed, Ziheng Wang, Jeon Lee, Rafe McBeth, Michael Skinner, Fenghua Tian, Ravi Thiagarajan, Lakshmi Raman

**Affiliations:** 1 Department of Pediatrics, Division of Pediatric Critical Care, Washington University School of Medicine, St. Louis, MO 63110, USA; neel.shah@wustl.edu; 2 Department of Pediatrics, Pediatrix Medical Group, Orem, UT 84057, USA; azfarhat@gmail.com; 3 Children’s Health Dallas, Dallas, TX 75201, USA; jefferson.tweed@childrens.com; 4 Department of Mechanical Engineering, The University of Texas at Dallas, Dallas, TX 75080, USA; zihengwang@utdallas.edu; 5 Department of Bioinformatics, University of Texas Southwestern, Dallas, TX 75390, USA; jeon.lee@utsouthwestern.edu; 6 Department of Radiation Oncology, University of Texas Southwestern, Dallas, TX 75390, USA; rafe.mcbeth@utsouthwestern.edu; 7 Department of Computer Science, The University of Texas at Dallas, Dallas, TX 75080, USA; skinner.ma@gmail.com; 8 Department of Bioengineering, University of Texas at Arlington, Arlington, TX 76019, USA; fenghua.tian@gmail.com; 9 Department of Cardiology, Boston Children’s Hospital, Boston, MA 02115, USA; ravi.thiagarajan@cardio.chboston.org; 10 Department of Pediatrics, Division of Pediatric Critical Care, University of Texas Southwestern, Dallas, TX 75390, USA

**Keywords:** ECMO, neurologic injury, brain injury, machine learning, neural networks, pediatrics

## Abstract

Brain injury is a significant source of morbidity and mortality for pediatric patients treated with Extracorporeal Membrane Oxygenation (ECMO). Our objective was to utilize neural networks to predict radiographic evidence of brain injury in pediatric ECMO-supported patients and identify specific variables that can be explored for future research. Data from 174 ECMO-supported patients were collected up to 24 h prior to, and for the duration of, the ECMO course. Thirty-five variables were collected, including physiological data, markers of end-organ perfusion, acid-base homeostasis, vasoactive infusions, markers of coagulation, and ECMO-machine factors. The primary outcome was the presence of radiologic evidence of moderate to severe brain injury as established by brain CT or MRI. This information was analyzed by a neural network, and results were compared to a logistic regression model as well as clinician judgement. The neural network model was able to predict brain injury with an Area Under the Curve (AUC) of 0.76, 73% sensitivity, and 80% specificity. Logistic regression had 62% sensitivity and 61% specificity. Clinician judgment had 39% sensitivity and 69% specificity. Sequential feature group masking demonstrated a relatively greater contribution of physiological data and minor contribution of coagulation factors to the model's performance. These findings lay the foundation for further areas of research directions.

## 1. Introduction

The use of Extracorporeal Membrane Oxygen (ECMO) in children has increased significantly over the last two decades [1,2]. Despite advances in ECMO science, the incidence of ECMO-related brain injuries remains high [3]. For children supported with ECMO who develop brain injury, survival is greatly reduced. Survivors with brain injury also have poor developmental and functional outcomes [4,5,6,7,8].

The etiology of brain injury is likely multifactorial. The severity of pre-ECMO illness and underlying comorbidities can predispose certain patients to develop an injury. Thromboembolic and hemorrhagic complications associated with extracorporeal support also contribute to injury risk. The extraordinary scope of data associated with an ECMO run has presented a challenge in elucidating the factors that most significantly contribute to brain injury risk and have limited development of a predictive model. This is partly due to the complex interactions between clinical and mechanical variables that limit the ability of traditional statistical methods to decipher causative factors or to generate an accurate prediction model. Existing research focuses on evaluating singular distinct elements, such as underlying illness, coagulation abnormalities, anticoagulation management, or markers of end-organ perfusion as factors associated with brain injury [9,10,11,12]. Many published models are limited to either pediatric or neonatal patients and have a narrow set of ECMO indications [13,14]. Prior studies do not take into consideration the temporal and dynamic element of clinical events that may play a large role in the genesis of brain injury, and few explore which variables may be predictive of injury without the bias of pre-selecting variables of interest.

Machine learning is a form of artificial intelligence that employs algorithms to elucidate patterns in an iterative manner, optimizing tasks such as prediction. The strength of neural networks lies in the ability to combine multiple factors, analyze their influence on each other, and incorporate temporal relationships in a model [15]. We hypothesized that neural network models utilizing temporal clinical data from prior and during the ECMO run in a large pediatric and neonatal ECMO population would predict radiographic brain injury. We further hypothesized that iterative feature masking would help to discriminate factors associated with the predictive ability of the model, and may guide future research avenues to mitigate neurological injuries. The overall purpose of the study is to gain some mechanistic insight that allows hypothesis generation for future studies into ECMO-related neurological injuries.

## 2. Materials and Methods

### 2.1. Study Oversight

This single-center observational cohort study was conducted in accordance with the ethical standards of the Committee for the Protection of Human Subjects at both University of Texas Southwestern Medical Center and Children’s Health. The institutional review board (IRB) at the University of Texas Southwestern Medical Center approved the parent study in 2015, and modification to include data for our study was approved in March of 2018. The need for consent was waived due to the retrospective nature of the study and deidentification of data.

### 2.2. Patients

**Figure 1:**
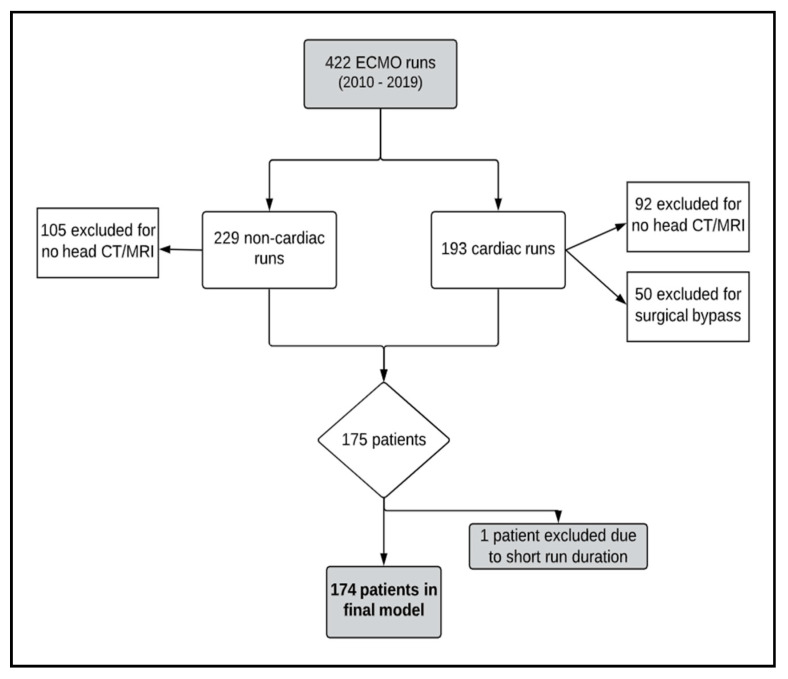
Schematic outlining patients identified, excluded from, and included in the study.

We queried our institutional ECMO database to identify children supported on ECMO in our tertiary pediatric and cardiac intensive care units between 1 January 2010 and 31 June 2019. As in Figure 1, a total of 422 ECMO runs occurred in that time period. All patients who received ECMO during this time for any indication were eligible. This included patients who were cannulated in the setting of extracorporeal cardiopulmonary resuscitation (ECPR). We excluded patients missing advanced neuroimaging in the form of either intra ECMO or CT or post-ECMO brain MRI/CT, as diagnosis of neurological injury was made by advanced neuroimaging. A small subset of patients died before imaging was performed; however, if they had head CTs prior to their death they were included in the analysis.

We also excluded cardiac bypass recipients since variable data from the operating room was not available. Due to imaging occurring post-ECMO, thus any clinical change that increased injury risk or injury itself that occurred during bypass would falsely be attributed to the ECMO run. No patients were lost to follow-up; all data were obtained within the hospitalization. After selection, 174 patients remained, with more than 35,000 ECMO hours and 1.4 million data points across 35 variables.

### 2.3. Brain Injury and Neuroimaging

The primary outcome was radiographic evidence of moderate to severe ischemic or hemorrhagic brain injury as categorized on computerized tomography (CT) or magnetic resonance imaging (MRI). Radiologic studies were either performed during ECMO or post-decannulation. MRIs were typically performed within a week of decannulation for patients supported with ECMO for non-cardiac indications. Head CT scans during ECMO were performed for acute clinical concern at the discretion of the primary ECMO physician. For patients who had a CT during ECMO as well as additional imaging (CT or MRI) post ECMO decannulation, brain imaging performed following ECMO was used for identifying brain injury. All head imaging was reviewed by a pediatric neuroradiologist blinded to clinical information. A random sample of ten images was reviewed by an independent neuroradiologist, who was blinded to imaging scores determined by the initial review. Scores assigned by both radiologists were reviewed for concordance. Cohen’s Kappa statistic was 0.82, indicating near-perfect agreement between the reviewers.

The diagnostic accuracy of these modalities in hemorrhagic brain injury [16] and ischemic brain injury has been reported previously [16,17,18]. Imaging was used instead of neurodevelopmental outcomes to allow the inclusion of patients who expired on ECMO or died prior to hospital discharge. In this population, neuroimaging has been correlated with neurodevelopmental outcomes [19,20,21].

### 2.4. Data Collection and Sources

Data were collected starting at 24 h prior to ECMO and for the entire duration of ECMO support. All information was extracted from the Epic™ electronic health records system. Any aberrant or missing data points prompted a manual review of the patient’s chart for reconciliation. No patients had missing data points in the final dataset. Physiologic and medication infusion data were imported hourly into the EHR automatically from physiologic monitors and manually verified by nursing staff. Laboratory values were inputted into the system automatically or by laboratory personnel; these were available at varying intervals based on ordered frequency. ECMO pump factors were available on an hourly basis for the duration of the run, as recorded by the ECMO specialist.

### 2.5. Data Organization

We extracted 35 variables and organized them into six distinct clinical groups as seen in Table A1. Physiologic data included systolic blood pressure, diastolic blood pressure, mean arterial blood pressure, heart rate, oxygen saturation (SpO2), and temperature (Celsius). Blood pressure measurements were based off of invasive arterial blood pressure catheters. Acid/Base homoeostasis factors collected were arterial pH, arterial partial pressure of carbon dioxide (PCO2), arterial partial pressure of oxygen (PaO2) and base excess. Surrogates of end-organ perfusion were collected including alanine aminotransferase (ALT), aspartate aminotransferase (AST), creatinine, lactate, and bilirubin. Serum glucose data were also collected. Markers of coagulation and hemolysis were collected and included International Normalized Ratio (INR), prothrombin time (PTT), platelet count, fibrinogen, hemoglobin, free hemoglobin, and heparin drip infusion rates (units/kg/h). Vasoactive data included: epinephrine, dopamine, norepinephrine, and milrinone infusion rates all as micrograms/kg/min; vasopressin infusion rate in units/kg/h; these values were also used to calculate a vasoactive infusion score (VIS) [22]. Lastly, we collected ECMO mechanical data including pressures pre-oxygenator, post-oxygenator, pressure at the volume sensor, measured flow (mL/kg/h), oxygenator FiO2, and sweep (L/min). All available variables were inputted with no prior preselection to minimize underlying bias factoring into the model, with the exception of age and diagnosis—these were excluded to limit leakage to the model.

### 2.6. Machine Learning Methods

**Figure 2:**
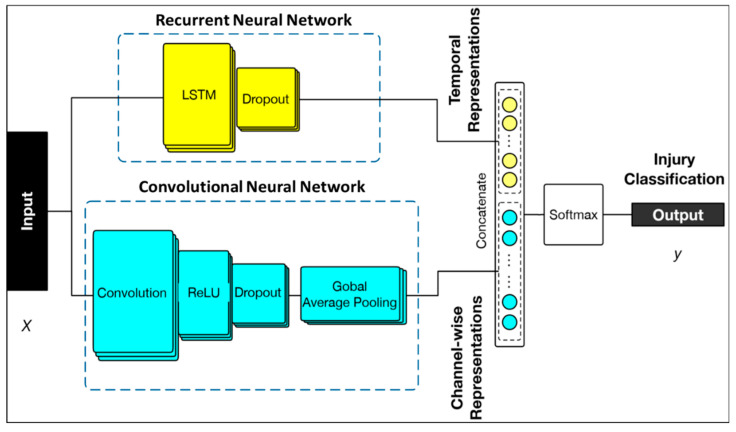
Machine learning architecture of the model used in the study. Hybrid neural network constructed of a recurrent neural network (Long Short-term Memory followed by dropout layer) and convolutional neural network (convolutional, Rectified linear unit (ReLU) activation unit and dropout and global pooling layers). Inputs were variables listed, with outputs being neurological injury classification.

Figure 2 shows the architecture of the machine learning algorithm. The network input is the raw multi-dimensional clinical data and the output is the predicted label of injury. The resulting neural network was composed of two main components in parallel, a convolutional network and a recurrent network.

The Convolutional Neural Network (CNN) consisted of a 1-dimensional convolutional layer with 16 filters and a kernel size of 5. It utilized rectified linear (ReLU) activation factors as well as a batch normalization layer. A global average pooling layer was then applied to generate the spatial information in each dimension. The Recurrent Neural Network (RNN) consisted of a Gated Recurrent Unit (GRU) with 16 hidden units. Two sets of abstract features extracted from both the CNN and RNN were concatenated into a new fully connected layer. Finally, the hybrid neural network and new linked layer generated an output for estimating radiographic brain injury probability through a softmax layer for classifications. The hybrid model was compared to individual RNN, CNN and a simple LTSM and demonstrated improved accuracy and F1 score.

We implemented our approach using Keras with Tensorflow 1.9 software (Mountain View, CA, USA) as our back-end platform [23]. Temporal samples were extracted at regular intervals. The deep learning model was trained by minimizing the cross-entropy loss using the Adam optimizer [24]. Hyper-parameter settings were 300 learning epochs and a stochastic gradient descent with initial learning rate of 0.0001. To obtain a robust process of modeling, all training samples were randomly shuffled in each epoch before feeding into the model. We performed all experiments on a remote server (UTSouthwestern BioHPC–Dallas, TX, USA) and utilized a computing cluster equipped with a NVIDIA Tesla P100 GPU and 72 CPUs with 256 GB memory(Santa Clara, CA, USA).

To evaluate the model performance and capability for generalizing new samples, we used 5-fold cross-validation settings that consist of multiple rounds. For the i-th round (i = 1, …, 5), the data were randomly split into a subset (80%) for training the model and the other subset (20%) was hold out for testing. In order to reduce any evaluation bias, the results in the testing sets over five rounds were averaged to give an estimate of model performance. We further limited the model to the first 48 and 120 h (including the 24 h prior to ECMO cannulation) to evaluate how the duration of recording would affect the ability to predict radiographic brain injury.

To improve post-hoc interpretability of our model, we removed groups of variables (specified in methods) and observed the overall effect on model accuracy. This was done by a machine learning technique known as feature masking, with the goal to elucidate the importance of variable groups to the models accuracy [25]. Following removal of each group, the model was once again validated with k-fold cross validation with the same 5-fold model evaluation setting, and the overall average accuracy and resulting confusion matrices were reported.

### 2.7. Clinician Suspicion for Injury

We reviewed all patients’ medical records to ascertain if brain injury was suspected during the ECMO run based on clinical assessment, prior to obtaining any imaging. This review included physician and advanced practice provider notes, subspecialty consults, and all requested imaging studies. We compared these findings with the results of imaging obtained following ECMO.

### 2.8. Logistic Regression

For each variable, four features were extracted over the initial 48 h: minimum, maximum, median value and slope. Data were limited to 48 h, as it was not possible to compare data over uneven time series of each patient using conventional statistical methods. We further normalized the data to account for irregular sampling intervals. Univariate analyses were performed to identify features that were associated with brain injury (Wilcoxon rank-sum test, *p* < 0.01). Five features were identified: Base Excess Maximum, Glucose minimum, glucose median, pCO2 minimum, and pH minimum. They were used as inputs to train a multivariate logistic regression predictor and the performance was evaluated through using k-fold cross validation (5 subsample groups).

## 3. Results

### 3.1. Patients

The final model included 174 patients. Patient characteristics are in Table 1 and Table 2. Fifty-one percent had brain injury. Of the injured patients, fifty-eight percent had a predominantly ischemic injury and forty-two percent had a predominantly hemorrhagic injury. There were no statistically significant differences between patients with and without brain injury with regards to ECMO indication, ECMO type, or gender. Differences existed in the age groups as seen in Table 2.

All neonates in this cohort were full-term. The median time from ICU admission to ECMO cannulation was one day. All patients received a heparin bolus prior to the initiation of ECMO, and all patients were managed with centrifugal pumps. Peripheral vessels were used for ECMO cannulation in 125 patients, and central cannulation was used in 49 patients. 

### 3.2. Injury Prediction Based on Neural Networks

**Figure 3:**
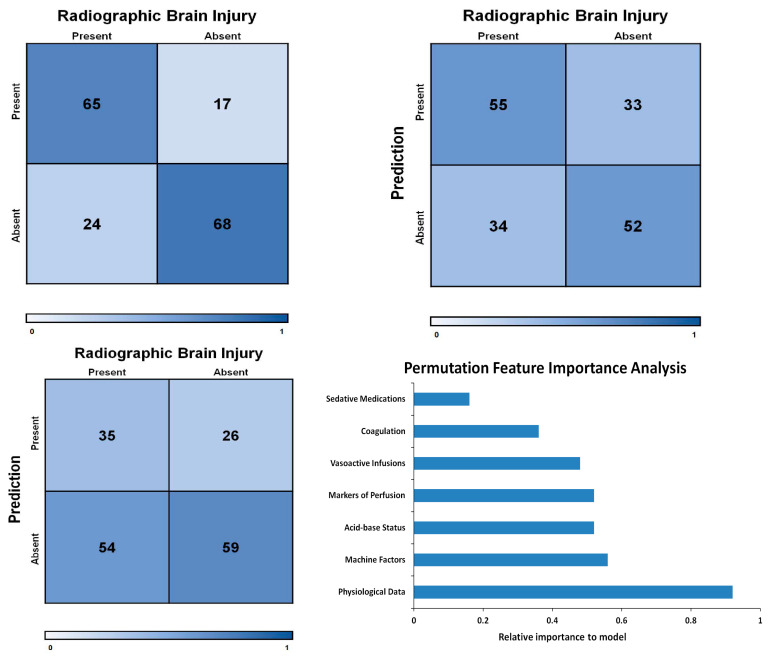
(**a**) Demonstrates model’s confusion matrix. (**b**) Demonstrates logistic regression model’s confusion matrix. (**c**) Matrix based on clinician suspicion for occurrence of injury. (**d**) Results of permutation feature importance, and relative importance for each variable group.

The developed models included over 35,000 clinical hours on ECMO and 1.4 million data points across 35 variables. Figure 3a shows the resulting confusion matrix. Radiographic brain injury was predicted with an Area Under the Curve (AUC) of 0.76. The model demonstrated 73% sensitivity for predicting radiographic brain injuries in our cohort. The model’s specificity was 80%, indicating good discrimination. The positive predictive value was 78% and the negative predictive value was 75%. The model’s F1 score, a measure of precision and recall, was 0.76.

Feature masking was performed to help elucidate which factors played a greater role in the model’s predictive ability. Physiological data had the highest impact on model accuracy. Other category groups had little to no impact on the model, as shown in Figure 3d and Table A2. Detailed model results are in the Appendix A, in Table A3 and Figure A2a–f.

**Figure A1:**
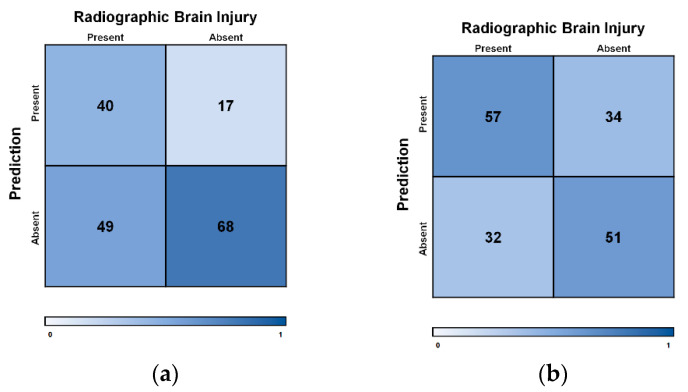
(**a**) Demonstrates model’s confusion matrix when limited to only initial 48 h of data. (**b**) Demonstrates model’s confusion matrix when limited to only initial 120 h of data.

**Figure A2:**
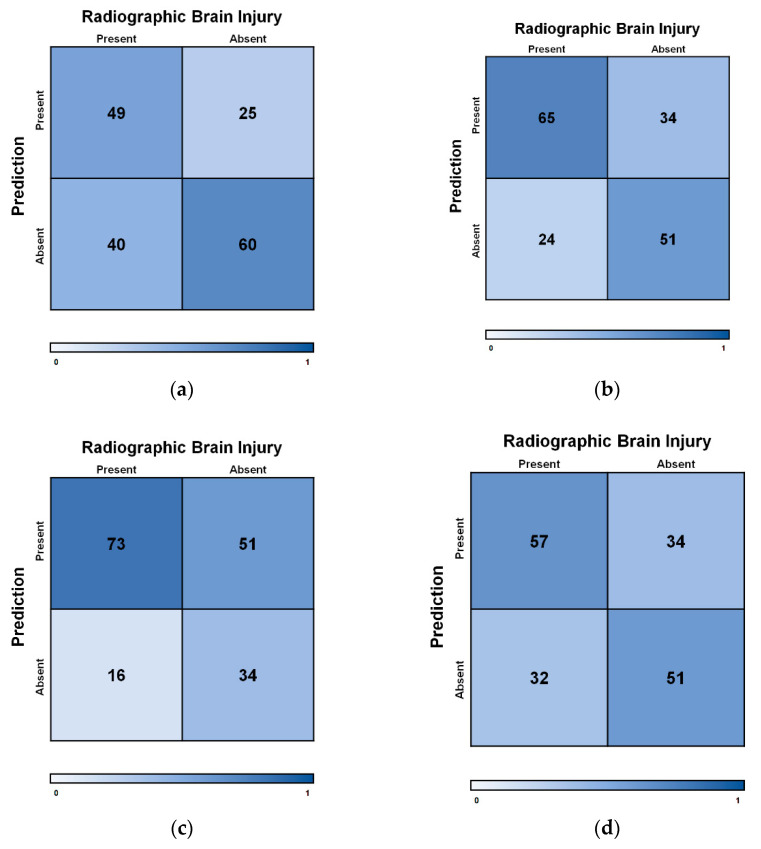

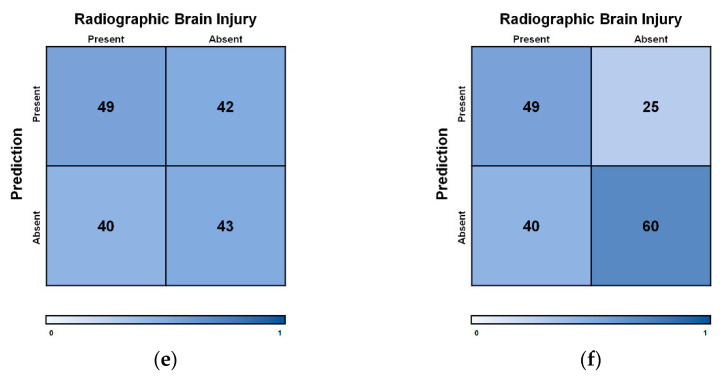
(**a**) Demonstrates model’s confusion matrix when after removal of acid-base factors during feature masking. (**b**) Demonstrates model’s confusion matrix when after removal of coagulation factors during feature masking. (**c**) Demonstrates model’s confusion matrix when after removal of ECMO machine factors during feature masking. (**d**) Demonstrates model’s confusion matrix when after removal of markers of perfusion during feature masking. (**e**) Demonstrates model’s confusion matrix when after removal of vital signs during feature masking. (**f**) Demonstrates model’s confusion matrix when after removal of vasoactives during feature masking.

Following this, the model was restricted to the first 48 or 120 h of data, including the pre-cannulation period. The resulting models demonstrated poor accuracy based on AUC values; Figure A1 shows the results.

### 3.3. Clinician Suspicion for Injury

Injury was suspected by the clinical team in 61 of the 174 patients (35%), all of whom underwent intra-run head CTs. Of these patients, 35 (57%) demonstrated moderate to severe radiographic brain injury. 26 demonstrated no significant injury. To encompass the progressive nature and possible radiographic delay of brain injury, a true negative was counted as a patient who was scanned intra-run and was not found to have an injury post-run. Clinical suspicion of injury had a sensitivity of 39% and specificity of 69%. The positive predictive value was 57% and the negative predictive value was 52% (Figure 3c and Table A3).

### 3.4. Injury Prediction Based on Conventional Methods

A total of 5 of the 35 variables within the first 48 h were associated with brain injury by univariate analysis. The multivariate logistic regression conducted on these five features demonstrated an AUC of 0.61, with a 62% sensitivity and 61% specificity (Figure 3b and Table A3).

## 4. Discussion

This study describes a novel approach to understand ECMO-related radiographic brain injury based on the comprehensive incorporation of ECMO-run clinical factors into a machine learning model. Despite incorporating a heterogeneous population with varying ECMO indications and clinical courses, the model displayed fair performance metrics as evidenced by an AUC of 0.76 and an F1 score of 0.76. The model had a positive predictive value (PPV) of 78%. Clinician judgment in patients suspected of injury had an overall accuracy of 54%, with a PPV of 57%. A traditional multivariate logistic regression model demonstrated a predictive accuracy of 61% and a PPV of 63%. Given the historically opaque cause of ECMO related neurologic injury, lessons learned from this work set the framework for prospective work with a larger more granular dataset.

The use of feature masking allowed us to run the model repeatedly while systematically removing groups of variables [26,27]. The technique provided an indirect assessment of different variables’ significance to the model. Hemodynamics, including perturbations in heart rates and blood pressures, had the greatest association with the prediction of occurrence of injury. Markers of coagulation and hemolysis, traditionally thought to significantly contribute to neurologic injury, had minimal impact on the model’s accuracy—consistent with some recent reports in the literature [10]. Acid-base status, vasoactive drug dosage, and ECMO machine settings had an insignificant effect on injury incidence. While cause and effect are unknown, this analysis indicates attention to hemodynamic parameters may be a fruitful direction for future research.

In contrast to the model’s 73% sensitivity and 80% specificity, clinician judgment had an overall accuracy of 54% with sensitivity of 39% and specificity of 69%. The model had a positive predictive value of 78%, opposed to a clinician PPV of 57%. Within this group, nearly half of the patients not clinically suspected to have an injury ended up with radiographic evidence of injury following their ECMO run. For an injury with grave consequences, missing a significant number of injuries until imaging following ECMO delays the ability to mitigate the effects of an injury. This demonstrates the need for better predictive modeling. The traditional multivariate logistic regression model was only marginally better, with a predictive accuracy of 61%, 62% sensitivity and 61% specificity.

Previously published clinical applications for neural networks include predicting mortality in the critical care setting [28,29], predicting the occurrence of severe sepsis [30,31], and identifying optimal approaches to treat infection [32]. These studies demonstrate the applicability of neural networks to questions in clinical medicine and utilized similar approaches to examine complex clinical relationships. To our knowledge, there are no previously developed tools that analyze brain injury on ECMO.

There are other ECMO-related outcomes for which predictive tools have been designed. An example is the P-PREP (Pediatric Pulmonary Rescue with ECMO Prediction) score. This score aimed to predict mortality at the time of ECMO initiation for children with respiratory failure (AUC 0.66) [14]. Such tools, however, use logistic regression to identify variables that contribute to the outcome. This approach presupposes a linear, non-complex relationship between input and outcome and thus cause and effect [33]. A machine learning approach provides more comprehensive and inclusive insight into these types of data.

Our institution adopted a unique practice of routine post-ECMO head imaging of non-cardiac patients when clinically stable—regardless of overt neurologic indication. This allowed for the identification of injuries that may have otherwise not been detected and may explain the higher incidence of brain injury within our cohort. Furthermore, patients treated with ECMO for cardiac indications were often not imaged if injury was not suspected. Limiting the analysis to the subset of patients with imaging, and not the entire cohort of patients, allowed us to provide the model with a defined outcome. The exclusion of cardiac bypass patients helped avoid potential confounders or brain injuries that may have been sustained prior to ECMO. Additionally, we excluded patients with known brain injury or abnormal imaging pre-ECMO to limit further confounders. Overall, our patient population is representative of pediatric ECMO-supported patients at large tertiary care centers.

### Limitations

A neural network’s predictive ability is improved with larger datasets. For a pediatric ECMO study, our dataset is large. In machine learning, 174 patients represent a small dataset, even if compensated for by the number of data points available per patient. Our dataset is susceptible to biases expected from a model trained and validated on patients from a single center, possibly affecting the model’s external validity. A model analyzing a large, more homogenous population will have better accuracy and external validity. We were also limited by utilizing only neuroimaging as our outcome, which may not correlate as well with functional neurodevelopmental outcomes in some patient groups. Our outcome of interest was radiographic neurologic injury, but by selecting only patients with post ECMO imaging, we may have introduced a selection bias. Nevertheless, based on other work in our institution, we do know that imaged and non-imaged patients are very similar from a demographic and outcome characteristic standpoint.

The lack of portable CTs in our center may have resulted in missing some injured patients if death occurred before decannulation, or if the patient was too unstable for transport. Excluding cardiac bypass patients, although undertaken due to missing clinical information, adds additional selection bias to this study. Any selection bias can result in erroneous conclusions including overestimated positive predictive values.

The retrospective review of the medical record may not be an accurate representation of true clinician suspicion, since some decisions may not be wholly captured in the record, and selection bias may exist. Finally, despite the feature masking performed, the inherent nature of neural networks prevents accurate interrogation of specific layers and weights generated by the model. This is especially true as features were analyzed in groups, and there may be covariance between variables that may over- or underestimate the contribution of any given feature group.

## 5. Conclusions

The etiology of brain injury on ECMO is complex and multifactorial. In this paper, we present a neural network’s predictive ability to identify radiographic brain injury, compared to both traditional statistical methods and clinical suspicion. We utilize feature masking to demonstrate that perturbations in hemodynamic parameters had the highest impact on the ability of our model to predict radiographic brain injury. Coagulation factors, which traditionally have been suspected to precipitate brain injury, had limited effect. Future application of this prediction model using more granular data in real-time will help improve our understanding of neurological injury on ECMO.

In a field that has been historically difficult to crack, these findings lay the foundation for further areas of research directions. This work also illustrates the need for the development of a real-time risk prediction model in order to advance the field towards the active optimization of treatments to mitigate these risks.

## Figures and Tables

**Table 1:** Demographic and clinical characteristics of patients included in the study.

Demographics of Patients in Database		
**Age**	Range	0–18 years
	Median (IQR)	10 months (6 years)
**Gender**	Male	92 (53%)
	Female	82 (47%)
**Weight (kg)**	Range	1.9–132
	Median (IQR)	5.9 (14.2)
**ECMO Type**	Venoarterial	106 (61%)
	Venovenous	68 (39%)
**ECMO Primary Indication**	Cardiac	49 (28%)
	Non-cardiac	125 (72%)
**ECMO Run Length (hours)**	Range	14–985
	Median (IQR)	129 (165)
**Injury Status**	Injury	89 (51%)
	No injury	85 (49%)

**Table 2:** Characteristics of patients in the injury vs no injury groups.

Patient Characteristics			
	Neurological Injury	No Neurological injury	*p*
**Primary ECMO indication *(total n: 174)***			
Non-cardiac	64	61	0.85
Cardiac	25	24	0.50
**Initial mode of ECMO *(total n: 174)***			
Venoarterial	61	45	0.12
Venovenous	28	40	0.15
**Gender *(total n: 174)***			
Male	49	43	0.53
Female	40	42	0.83
**Age *(total n: 174)***			
Neonate (0–30 days)	38	32	0.47
Infant (1–12 months)	8	21	0.02 *
Child (1–12 years)	35	19	0.03 *
Adolescent (>12 years)	8	13	0.28
Survived to hospital discharge	76%	72%	0.74

* Statistically Significant at 95% Confidence Interval.

## Appendix A

**Table A1:** Breakdown of which variables were included within each group.

Variable Group	Included Variables						
Acid-Base	Arterial pH	Arterial CO2 (pCO2)	Arterial Oxygen (pO2)	Base Excess			
Coagulation	International Normalized Ratio (INR)	Prothrombin time (PTT)	Platelet count	Fibrinogen	Hemoglobin	Free Hemoglobin	Heparin drip infusion rate (units/kg/h)
Machine Factors	Pre-oxygenator pressure	Post-oxygenator pressure	Pressure at Volume sensor	Measured flow (mL/kg/h)	Oxygenator FiO2	Sweep (L/min)	
Markers of End-Organ Perfusion	Alanine aminotransferase (ALT)	Aspartate aminotransferase (AST)	Creatinine	Lactate	Bilirubin	* Glucose	
Vasoactive Medications	Epinephrine (mcg/kg/min)	Dopamine (mcg/kg/min)	Norepinephrine (mcg/kg/min)	Milrinone (mcg/kg/min)	Vasopressin (units/kg/h)	Vasoactive Infusion Score (VIS)	
Physiological Data	Systolic blood pressure	Diastolic blood pressure	Mean blood pressure	SpO2	Heart Rate	Temp (°C)	

* Glucose not included in this group for permutation feature importance.

**Table A2:** Magnitude of variable group importance to final model predictions following feature masking.

Data Group	Magnitude of Importance
Acid-base	0.52
Coagulation	0.36
Machine factors	0.56
Markers of perfusion	0.52
Vasoactives	0.48
Physiological data	0.92

**Table A3:** Detailed statistical results of all assessed models in study.

Model	Sensitivity	Specificity	PPV	NPV	+LR	−LR	AUC
Complete Model	73%	80%	78%	75%	3.65	0.38	0.76
**Alternate assessments:**							
Clinician suspicion	39%	69%	57%	52%	1.29	0.87	0.54
Logistic Regression	62%	61%	63%	60%	1.59	0.62	0.61
**Permutation feature importance and data-groups excluded:**							
Acid-base	55%	71%	66%	60%	1.87	0.64	0.62
Coagulation	73%	60%	66%	68%	1.83	0.45	0.67
Machine factors	82%	40%	59%	68%	1.37	0.45	0.61
Markers of perfusion	64%	60%	63%	62%	1.60	0.60	0.62
Vasoactives	55%	70%	66%	60%	1.87	0.64	0.63
Physiological data	55%	51%	54%	52%	1.11	0.90	0.53

## References

[1] Extracorporeal Life Support Organization-ECMO and ECLS > Registry > Statistics > International Summary. https://www.elso.org/Registry/Statistics/InternationalSummary.aspx.

[2] Jenks C.L., Raman L., Dalton H.J. (2017). Pediatric Extracorporeal Membrane Oxygenation. Crit. Care Clin..

[3] Lorusso R., Gelsomino S., Parise O., Di Mauro M., Barili F., Geskes G., Vizzardi E., Rycus P.T., Muellenbach R., Muellenbach R. (2017). Neurologic Injury in Adults Supported With Veno-venous Extracorporeal Membrane Oxygenation for Respiratory Failure: Findings From the Extracorporeal Life Support Organization Database. Crit. Care Med..

[4] Polito A., Barrett C.S., Wypij D., Rycus P.T., Netto R., Cogo P.E., Thiagarajan R.R. (2013). Neurologic complications in neonates supported with extracorporeal membrane oxygenation. An analysis of ELSO registry data. Intensive Care Med..

[5] Madderom M.J., Reuser J.J.C.M., Utens E.M.W.J., van Rosmalen J., Raets M., Govaert P., Steiner K., Gischler S.J., Tibboel D., van Heijst A.F.J. (2013). Neurodevelopmental, educational and behavioral outcome at 8 years after neonatal ECMO: A nationwide multicenter study. Intensive Care Med..

[6] Boyle K., Felling R., Yiu A., Battarjee W., Schwartz J.M., Salorio C., Bembea M.M. (2018). Neurologic Outcomes After Extracorporeal Membrane Oxygenation: A Systematic Review. Pediatr. Crit. Care Med..

[7] Bembea M.M., Felling R.J., Caprarola S.D., Ng D.K., Tekes A., Boyle K., Yiu A., Rizkalla N., Schwartz J., Everett A.D. (2020). Neurologic Outcomes in a Two-Center Cohort of Neonatal and Pediatric Patients Supported on Extracorporeal Membrane Oxygenation. ASAIO J..

[8] Waitzer E., Riley S.P., Perreault T., Shevell M.I. (2009). Neurologic Outcome at School Entry for Newborns Treated With Extracorporeal Membrane Oxygenation for Noncardiac Indications. J. Child Neurol..

[9] Barbaro R.P., Bartlett R.H., Chapman R.L., Paden M.L., Roberts L.A., Gebremariam A., Annich G.M., Davis M.M. (2016). Development and validation of the neonatal risk estimate score for children using extracorporeal respiratory support. J. Pediatr..

[10] Anton-Martin P., Raman L., Thatte N., Tweed J., Modem V., Journeycake J. (2017). Pre-ECMO Coagulopathy does not Increase the Occurrence of Hemorrhage during Extracorporeal Support. Int. J. Artif. Organs.

[11] Park S.J., Kim S., Kim J.B., Jung S.-H., Choo S.J., Chung C.H., Lee J.W. (2014). Blood lactate level during extracorporeal life support as a surrogate marker for survival. J. Thorac. Cardiovasc. Surg..

[12] Kolodziej A., Burchett A., Tribble T., Grigorian A.Y., Guglin M. (2017). Lactic Acid Is the Most Important Factor Predicting Survival on VA ECMO. J. Heart Lung Transplant..

[13] Akin S., Caliskan K., Soliman O., Muslem R., Guven G., van Thiel R.J., Struijs A., Gommers D., Zijlstra F., Bakker J. (2020). A novel mortality risk score predicting intensive care mortality in cardiogenic shock patients treated with veno-arterial extracorporeal membrane oxygenation. J. Crit. Care.

[14] Bailly D.K., Reeder R.W., Zabrocki L.A., Hubbard A.M., Wilkes J., Bratton S.L., Thiagarajan R.R. (2017). Development and Validation of a Score to Predict Mortality in Children Undergoing Extracorporeal Membrane Oxygenation for Respiratory Failure: Pediatric Pulmonary Rescue With Extracorporeal Membrane Oxygenation Prediction Score. Crit. Care Med..

[15] Wang Z., Majewicz Fey A. (2018). Deep learning with convolutional neural network for objective skill evaluation in robot-assisted surgery. Int. J. Comput. Assist. Radiol. Surg..

[16] Kidwell C.S., Chalela J.A., Saver J.L., Starkman S., Hill M.D., Demchuk A.M., Butman J.A., Patronas N., Alger J.R., Latour L.L. (2004). Comparison of MRI and CT for Detection of Acute Intracerebral Hemorrhage. JAMA.

[17] Lidegran M.K., Mosskin M., Ringertz H.G., Frenckner B.P., Lindén V.B. (2007). Cranial CT for diagnosis of intracranial complications in adult and pediatric patients during ECMO: Clinical benefits in diagnosis and treatment. Acad. Radiol..

[18] Chalela J.A., Kidwell C.S., Nentwich L.M., Luby M., Butman J.A., Demchuk A.M., Hill M.D., Patronas N., Latour L., Warach S. (2007). Magnetic resonance imaging and computed tomography in emergency assessment of patients with suspected acute stroke: A prospective comparison. Lancet.

[19] Parikh N.A. (2016). Advanced neuroimaging and its role in predicting neurodevelopmental outcomes in very preterm infants. Semin. Perinatol..

[20] Jose A., Matthai J., Paul S. (2013). Correlation of EEG, CT, and MRI Brain with Neurological Outcome at 12 Months in Term Newborns with Hypoxic Ischemic Encephalopathy. J. Clin. Neonatol..

[21] Slaughter L.A., Bonfante-Mejia E., Hintz S.R., Dvorchik I., Parikh N.A. (2016). Early Conventional MRI for Prediction of Neurodevelopmental Impairment in Extremely-Low-Birth-Weight Infants. Neonatology.

[22] McIntosh A.M., Tong S., Deakyne S.J., Davidson J.A., Scott H.F. (2017). Validation of the Vasoactive-Inotropic Score in Pediatric Sepsis. Pediatr. Crit. Care Med..

[23] Abadi M., Barham P., Chen J., Chen Z., Davis A., Dean J., Devin M., Ghemawat S., Irving G., Isard M. TensorFlow: A System for Large-Scale Machine Learning. https://www.usenix.org/system/files/conference/osdi16/osdi16-abadi.pdf.

[24] Bock S., Goppold J., Weiß M. An Improvement of the Convergence Proof of the ADAM-Optimizer. https://arxiv.org/abs/1804.10587.

[25] McWilliams C.J., Lawson D.J., Santos-Rodriguez R., Gilchrist I.D., Champneys A., Gould T.H., Thomas M.J., Bourdeaux C.P. (2019). Towards a decision support tool for intensive care discharge: Machine learning algorithm development using electronic healthcare data from MIMIC-III and Bristol, UK. BMJ Open.

[26] Leray P., Gallinari P. (1999). Feature Selection with Neural Networks. Behaviormetrika.

[27] Khemphila A., Boonjing V. Heart Disease Classification Using Neural Network and Feature Selection. Proceedings of the 2011 21st International Conference on Systems Engineering.

[28] Aczon M., Ledbetter D., Ho L., Gunny A., Flynn A., Williams J., Wetzel R. (2017). Dynamic Mortality Risk Predictions in Pediatric Critical Care Using Recurrent Neural Networks. arXiv.

[29] Perng J.-W., Kao I.-H., Kung C.-T., Hung S.-C., Lai Y.-H., Su C.-M. (2019). Mortality Prediction of Septic Patients in the Emergency Department Based on Machine Learning. J. Clin. Med..

[30] Le S., Hoffman J., Barton C., Fitzgerald J.C., Allen A., Pellegrini E., Calvert J., Das R. (2019). Pediatric Severe Sepsis Prediction Using Machine Learning. Front. Pediatr..

[31] Yee C.R., Narain N.R., Akmaev V.R., Vemulapalli V. (2019). A Data-Driven Approach to Predicting Septic Shock in the Intensive Care Unit. Biomed. Inform. Insights.

[32] Peiffer-Smadja N., Rawson T.M., Ahmad R., Buchard A., Georgiou P., Lescure F.-X., Birgand G., Holmes A.H. (2020). Machine learning for clinical decision support in infectious diseases: A narrative review of current applications. Clin. Microbiol. Infect..

[33] Jaimes F., Farbiarz J., Alvarez D., Martínez C. (2005). Comparison between logistic regression and neural networks to predict death in patients with suspected sepsis in the emergency room. Crit. Care.

